# Round the Hospitals

**Published:** 1921-03-19

**Authors:** 


### ROUND THE HOSPITALS.

The vacancy in the matronship at the Radcliffe Infirmary, Oxford, caused by the retirement of Miss Watt, which was advertised in our issue of January 22, has been filled by the appointment of Miss M. M. Biggar, sister of St. Thomas's Hospital. Miss Biggar's nursing experience extends over twelve years and is rich in its variety and scope. She trained in the Nightingale School of St. Thomas's from 1909 to 1912, and filled successively the posts of sister-in-charge of the gynæcological ward of Block VIII., containing some eighty beds for special cases, with an operating-theatre and a large tuberculosis out-patient department, assistant in Matron's Office, and various holiday posts, including that of assistant matron, housekeeper, and sister of the Nightingale Home. As honorary secretary of the London Centre of the College of Nursing she has done much to contribute to the success of the Centre and Club and to develop its organisation. The Radcliffe Infirmary is undoubtedly happy in its selection, and we offer our congratulations also to Miss Biggar, who should go far in the field which is open to her to cultivate. She will find her labours facilitated by the happy tone of loyalty and good comradeship set flowing by her predecessor.

The death of Lady Henry Somerset removes one of the foremost temperance reformers of our times. Her vigorous and successful propaganda work secured her the position of President of the World's Women's Christian Temperance Union. She was the first to introduce the industrial farm colony system for women victims of inebriety into this country, and her Home at Duxhurst proved not only a refuge for many wretched sufferers, but a centre for progressive study of the conditions most

**ROUND THE HOSPITALS**—(*continued*).

suitable for promoting cure and training workers to deal with this social evil. Lady Henry Somerset had for fifteen years a home for training workhouse children, and also a home for children received from the National Society for the Prevention of Cruelty to Children. Mr. T. P. O'Connor, M.P., writing of her in the *Daily Telegraph*, compares her dedication of herself to the sacred cause, young, beautiful, and very rich, to the devotion of Florence Nightingale when she went to Scutari. "There were none too lovely, none too vicious, none even too degraded to affright the valour of her hopefulness and love."

THE Nurses' Co-operation, in issuing its thirtieth report, puts on record the great loss sustained by the death of Sir Henry Burdett, "one of the founders of the Co-operation, to whom its business-like footing and much of its early success was due." The three fundamental rules of the company are recapitulated. They are designed to secure the Co-operation from any attempt to break it up, and to ensure that the income and property must be devoted solely towards the promotion of its objects, that no member shall participate in profits or assets, and that if it be ever dissolved its property must be transferred to some other institution having similar objects. The company is composed of about thirty-one members, whose exertions can never in any degree benefit themselves, but are solely directed towards the aim of securing for the nurses on the staff the fruits of their own labours. The nurses on the staff now number 447, of whom thirty-seven are asylum trained. They contribute a percentage of their fees, limited to 5 per cent., for those who joined before 1913, and to 7½ per cent. for later comers. The management is entrusted to a committee of seventeen, of whom ten are elected by the nurses. The Howard de Walden Nurses' Home and Club for the use of nurses on the staff, at 22 Langham Street, is now a very valuable asset. Besides this, the company holds cash in hand, investments, and shares amounting to £3,500, a necessary reserve, for in some years the current expenditure on the objects of the institution has exceeded the income. Last year the excess of income over expenditure was £232—a small margin considering the fact that the net fees paid over to nurses reached the total of £55,740. The Nurses' Co-operation has never issued a more thoroughly satisfactory report.

SINCE the issue of the College of Nursing scale of salaries (which recommends a minimum fee of 3½ guineas for the private nurse) the Committee of the Nurses' Co-operation has been considering the fees to be charged by its nurses, which were raised to £3 3s. some time ago. The question was submitted to a referendum of the nurses on the staff, the alternatives being to charge a fixed fee of £3 3s. as at present or to have a sliding-scale of £3 3s. to £4 4s. for ordinary cases, according to the circumstances of the patient. A substantial majority has voted for a sliding-scale, which will henceforth be adopted. It has, we understand, been found to work well by the visiting nurses of the Co-operation, and it is hoped that its application to the resident private nurse will meet both her needs and the financial position of the patient. The Co-operation nurses are fortunate in having as their representative on the Committee Miss Geraldine Bremner, an ardent advocate on behalf of the private nurse, who is also a member of the Council of the College of Nursing.

ON another page, under "Parliament and the Professions," College members will be interested to notice Mr. C. E. Leo Lyle's intervention in a question that concerns them asked by a Labour member relating to the Nation's Fund for Nurses. Mr. Lyle has shown his watchful interest in nursing affairs in many directions, and nurses are fortunate in having such a friend as a member of the House of Commons. He is Chairman of Queen Mary's Hospital for Nurses; he worked hard to get for nurses a just Registration Act, and we believe it was greatly due to his vigilant care that the pensions of disabled Army nurses were increased. When College members come to elect the new Council we hope that if, as is likely, they give one of their votes to an independent candidate, the Nurses' M.P., Mr. C. E. Leo Lyle, will be that candidate.

MISS C. E. A. THORPE, A.R.R.C., who, after seventeen years' service at the West-End Hospital for Nervous Diseases, is retiring from the matronship, has been the recipient of presentations from the nursing staff, dispenser, and female clerks, the members of the training-school, the Secretary, and the porters and domestic staff. Miss Thorpe is not retiring from the nursing world, for she is shortly opening, with a friend, a Home for Invalid Children and Adults, bedridden or otherwise, at Heathfield, between Eastbourne and Tunbridge Wells. The good wishes of all her staff, past and present, go with her in this new venture.

AT the Investiture held by the King on March 9, Miss Nora Fletcher, late Matron-in-Chief in France of the British Red Cross, was invested with the insignia of Commander of the Order of the British Empire, Civil Division. This is a distinction upon which Miss Fletcher's many friends heartily congratulate her. Since demobilisation Miss Fletcher has struck out a new line for herself, for she and her sister—also a trained nurse we understand—have opened and for the past two years conducted tea and luncheon rooms at 13 High Street, Kensington. They are called the Manhattan tea rooms and are frequently the centre of some very cheery nursing reunions.

A NUMBER of cordial letters in appreciation of the Infirmary matron's services have been received by the Clerk to the Portsmouth Guardians. The sisters and nurses of the Milton Infirmary, to the number

**ROUND THE HOSPITALS**—(*continued*).

of 776, protest strongly against the imputations cast on the matron's conduct of the institution in the course of the recent controversies. They record that they have found her to be just and devoted to the patients and the nursing staff under all circumstances. "A number of us, having known her for many years, have reason to be most grateful for every quality desirable in an ideal matron. We hold her in the greatest esteem, and are full of admiration and gratitude for her kindnesses during our training." Thirteen children in one ward and 300 patients have also sent letters of appreciation. The nurses are particularly disturbed at the suggestion that they have shown disloyalty towards their matron.

Bradford is doing well by its nurses. At the Royal Infirmary during the past year real progress took place in developing the nursing on modern lines as regards conditions of service. The Nurses' Hostel at Field House, which has proved its value in affording additional accommodation for the nursing staff, has been already outgrown, owing to the decision of the governors to introduce one day's leave in seven for every nurse. An annexe at the back of Field House has already been commenced, with a new large dining-room on the site of the conservatory, and it is hoped this annexe will be ready for occupation about the middle of the year. It is no temporary structure, but has been so planned as to form part of the new infirmary when this is erected. An interesting feature in connection with the Ladies' Committee attached to the Bradford Royal Infirmary was the organisation of a band of domestic workers last spring, in response to an urgent appeal from the Matron. These ladies gave efficient and much-appreciated help as long as it was necessary.

A very important branch of social service is carried out in American cities in connection with the dispensary system. It embraces the knowledge of each patient's environment, the following up of each case till recovery is completely established, and the provision of institutional treatment should it be required. The number of women now engaged as auxiliaries in this service is large and rapidly increasing. Among the medical social workers of New York there is a strong opinion that nursing education is essential for success. The fact of a woman being a nurse makes her message more authoritative, and in dealing with hospital cases and following out the doctor's treatment it is hard to see how a lay woman can be of real use. Nevertheless, in certain directions women with no nursing training are largely employed, and actually placed in the position of superintendent. At the Massachusetts General Hospital, of the thirty-two social workers only three are nurses. The difficulty, it appears, is to get women with hospital training who possess the thorough social training required in this work. It is objected there, as sometimes in England, that "the training of nurses is not designed to nurture qualities of mind indispensable to successful social work of a high order." The General Nursing Council in its recently issued Draft Rules for Training appears to hold that this is to some extent a true indictment.

The little deficit of £12 on last year's working of the Midwives' Institute has been covered by sums subscribed at and after the annual meeting. The decision to raise the subscription of London and Home County members to 7s. 6d. will, it is believed, suffice to cover the increase in expenditure. These members enjoy advantages from which those living at a distance are cut off, and will no doubt readily contribute the little extra which makes so great a difference in the smooth running of the Institute.

